# Using MRI to predict future adverse cardiac remodelling in a male mouse model of myocardial infarction

**DOI:** 10.1016/j.ijcha.2016.03.005

**Published:** 2016-03-16

**Authors:** Rachael E. Redgrave, Simon Tual-Chalot, Benjamin J. Davison, Elizabeth Greally, Mauro Santibanez-Koref, Jurgen E. Schneider, Andrew M. Blamire, Helen M. Arthur

**Affiliations:** a Institute of Genetic Medicine, Central Parkway, Newcastle University, Newcastle NE1 3BZ, UK; b Radcliffe Department of Cardiovascular Medicine, University of Oxford, BHF Experimental MR Unit, Roosevelt Drive, Oxford OX3 7BN, UK; c Institute of Cellular Medicine, Framlington Place, Newcastle University, Newcastle NE4 5PL, UK

**Keywords:** Myocardial infarction, Animal models of human disease, Remodelling, MRI

## Abstract

**Background:**

Mice are frequently used in research to examine outcomes of myocardial infarction (MI) and to investigate therapeutic interventions at an early pre-clinical stage. The MI model is generated by surgically occluding a major coronary artery, but natural variation in murine coronary anatomy can generate variable outcomes that will inevitably affect the accuracy of such investigations. The aim of this study was to use MRI to derive the most sensitive early variable that could be used to predict subsequent adverse cardiac remodelling in a male mouse model of MI.

**Methods:**

Using a longitudinal study design, heart structure and function were evaluated using cardiac MRI at one week following surgical MI to generate the early measurements and again at four weeks, when the scar had matured. The primary variables measured at week one were left ventricular volumes at end systole (LV-ESV) and at end diastole (LV-EDV), infarct size, LV-cardiac mass, and ejection fraction (EF).

**Results:**

Univariate and multiple regression analyses showed that LV-ESV at one week following MI could be used to accurately predict various parameters of adverse LV remodelling at four weeks post-MI. However, the highest correlation was between LV-ESV at one week following MI and LV-EDV at four weeks (r = 0.99; p < 0.0001), making LV-ESV at one week a valuable predictor variable of future adverse ventricular remodelling after MI.

**Conclusion:**

Using MRI to determine LV-ESV at an early stage following MI enables a more robust analysis of potential therapeutic interventions to ameliorate adverse cardiac remodelling.

## 1 Introduction

It is well established that myocardial infarction resulting from the occlusion of a major coronary artery leads to cardiomyocyte necrosis and initiates a wound healing response. The resultant combination of cardiomyocyte death, inflammation and fibrosis leads to a situation where non-contractile scar tissue gradually replaces the infarcted myocardial tissue. Furthermore, increased pressure/volume load on the non-ischaemic myocardium leads to cardiac hypertrophy in non-infarcted areas, increasing the functional impairment of the affected ventricle and contributing to additional adverse remodelling. In parallel, systemic neurohormonal activation also contributes to progressive, adverse ventricular remodelling and development of heart failure [1], [2], [3]. Many of these processes occur in a similar manner in rodents and humans, and consequently mouse models of MI have been widely used to understand the processes leading to adverse ventricular remodelling and to investigate therapeutic interventions in the pre-clinical setting. Studies on mice also offer the advantage of being able to target genes of interest using genetic tools (e.g. Cre/Lox) to investigate gene-specific functions in heart injury and repair.

Surgical occlusion of a major left ventricular coronary artery in a wild type mouse is frequently used to model myocardial infarction [4]. Usually the left anterior descending (LAD) artery is ligated, immediately below the left auricle. This occludes the vascular supply to much of the free wall of the left ventricle (LV), sparing the septum and most of the right ventricle. However, even in experienced hands, the microsurgery leads to variation in infarct size due to (i) natural variation in coronary anatomy between individuals, and (ii) sub-millimetre differences in the surgeon's placement of the suture over the LAD [5]. Therefore, improved methods of predicting adverse cardiac remodelling are required to evaluate therapeutic improvement within the context of this baseline variation. This goal is of prime importance because reported effective therapies in mouse models of myocardial infarction to date are rarely translated to the clinic [6] and poor experimental design of mouse studies may partly explain these disappointing outcomes. This research field urgently requires a study design that can incorporate natural variation in infarct size and can be used as a robust basis for investigating effective therapeutic interventions.

A range of studies have investigated baseline outcomes in mouse models of MI. For example, Shioura and colleagues showed that surgical ligation of the left main coronary artery in wild type (C57BL/6) male mice showed an initial drop in systolic function, followed by a transient improvement before further decline in both systolic and diastolic function that correlated with dynamic changes in foetal gene expression, characteristic of heart failure [7]. However, a major limitation of this study, and other similar studies that use conductance catheters to examine cardiac function, is that each analysis involves a terminal procedure, prohibiting longitudinal studies. In contrast, cardiac MRI provides a powerful technique to measure heart function under transient anaesthesia and is considered the “gold standard” permitting longitudinal studies to track cardiac function in individual animals [8]. Using this technique Protti and colleagues recently investigated adverse cardiac remodelling following surgical ligation of the LAD in wild-type (C57BL/6) mice [9]. They observed that left ventricular end diastolic volume (LV-EDV) at an early stage (2 days) following MI was predictive of the adverse cardiac remodelling outcome at 30 days and that infarcted mice fell into two groups (i) progression and (ii) non-progression, with non-progression associated with small infarct size. However, their study was limited to female mice and a growing body of evidence from animal studies suggests that females exhibit a different pattern of post-MI healing and left ventricular remodelling compared to males. In essence, there is less inflammation and increased reparative fibrosis during infarct healing in female mice leading to reduced LV remodelling compared with male mice [10], [11], [12], [13]. Also, in a clinical context, MI is more common in men than women [14], [15] so it is also important to determine robust predictive measures in male animal models. To address this issue we investigated longitudinal outcomes using MRI in a male mouse model of myocardial infarction to determine which early adverse events were best able to predict further progression of adverse remodelling and could be used to measure the benefit of therapeutic outcomes in future studies.

## 2 Methods

### 2.1 Mouse model of myocardial infarction

**Fig. 1:**
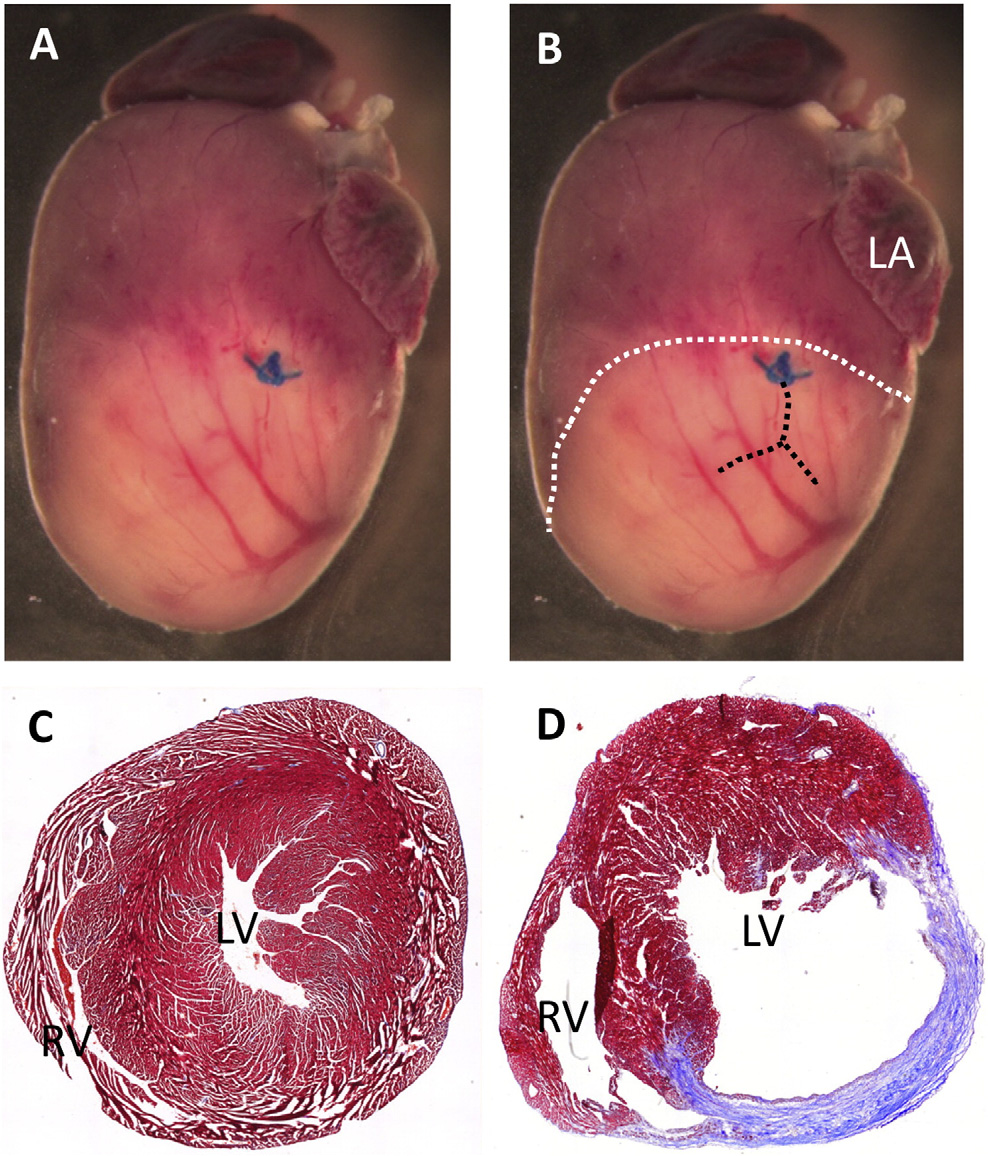
Mouse model of myocardial infarction by surgical ligation of the left anterior descending artery (LAD). A: Mouse heart 24 h following the occlusion of the LAD coronary artery. Note that in life, the left atrium is blood-filled and reaches just above the ligature. The ischaemic myocardium can be seen by the blanched area of myocardium extending to the apex. B: The position of the LAD coronary artery has been delineated with black lines and the boundary of the ischaemic area by dashed white line. C, D: Transverse sections of normal heart (C) and infarcted heart (D) taken 4 weeks after surgery and stained with Masson's trichrome to reveal extensive fibrosis (blue) associated with the dilated left ventricle in the infarcted heart. Abbreviations: LA, left atrium; LV, left ventricle; RV, right ventricle.

All in vivo procedures were conducted in accordance with the Guidance on the Operation of the Animals (Scientific Procedures) Act, 1986 (UK Home Office) and approved by the local ethics committee. Acute MI was created in 25 adult male C57BL/6 mice (12 to 14 weeks of age) using a procedure adapted from a published protocol [16]. Mice were pre-medicated with fentanyl/fluanisone (‘Hypnorm’, 0.4 ml/kg) to provide intra-operative analgesia and anaesthetised using isoflurane (3% isoflurane/97% oxygen). Anaesthesia was maintained using mechanical ventilation (Inspira Ventilator) following endotracheal intubation using 130–140 breaths per minute and 5 ml/kg tidal volume initially, increased to 7.5 ml/kg post-thoracotomy. Left-side thoracotomy was performed through the fourth intercostal space and the left anterior descending (LAD) coronary artery was ligated immediately below the left atria with a 7-0 prolene suture. Successful occlusion of the vessel was verified by immediate blanching of the left ventricular myocardium (Fig. 1). Mice in the sham group underwent left-side thoracotomy without LAD ligation. The chest was closed, mice were extubated and buprenorphine (Vetergesic, 0.05 mg/kg) was administered subcutaneously for post-operative analgesia. Mice were placed in a 33 °C incubator for 2 h immediately post-surgery to facilitate recovery and given soaked diet for 1 week so they did not have to stretch for food during the main period of thoracic wound repair.

### 2.2 Cardiac MRI

Magnetic resonance images were acquired at 1 week and 4 weeks following MI surgery using a horizontal bore 7.0T Varian microimaging system (Varian Inc., Palo Alto, CA, USA) equipped with a 12-cm microimaging gradient insert (40 gauss/cm). Animals were anaesthetised with isoflurane via a facemask and positioned on a custom built sled (Dazai Research Instruments, Toronto, Canada), with integrated electrocardiographic (via electrodes fastened to the shaved chest with conductive hydro-gel), respiratory (via a pneumatic pillow) and cutaneous temperature monitoring. An SA Instruments Inc. (Edison, NJ, USA) small animal system was used for physiological monitoring and gating. A 30 mm quadrature birdcage coil (Rapid Biomedical, GmbH) was used to transmit/receive the MR signal. Scout images were used to ensure that the short and long axes of the left ventricle were correctly orientated. Global cardiac function was measured using an ECG triggered, respiratory gated gradient echo (FLASH) cine MR sequence using the following imaging parameters: TE 1.36 ms, TR 5 ms, flip angle 15°, matrix 128 × 128, field of view 25.6 mm × 25.6 mm, and 4 averages. The cardiac cycle was imaged across a minimum of 20 phases in all animals. Sufficient contiguous short axis slices of 1 mm thickness were obtained to cover the whole left ventricle.

### 2.3 MR image analysis

**Fig. 2:**
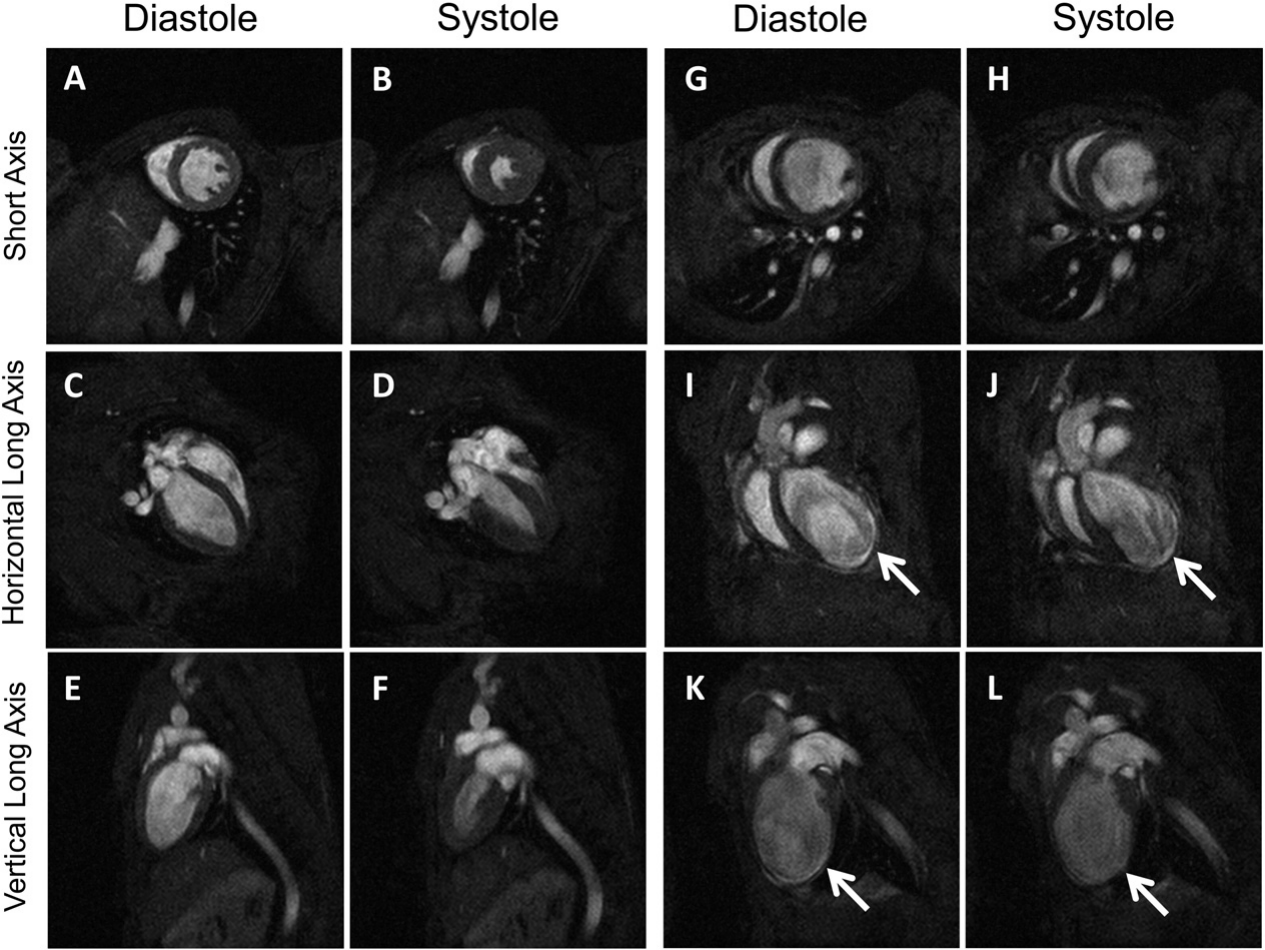
MRI images of normal and infarcted mouse hearts. A–F: MRI images of short, horizontal and long axis views of a normal mouse heart in systole and diastole. G–L: MRI images of short, horizontal and long axis views of an infarcted mouse heart 4 weeks after surgical ligation of the LAD. Note the enlarged left ventricle and thinned, akinetic area of the infarcted myocardium (arrows in I, J, K & L).

MR images confirmed expected cardiac changes (Fig. 2) and short axis images were analysed using Image J (NIH) by manually tracing epicardial and endocardial borders as previously published [8]. The area between the epicardial and endocardial borders was measured in all slices at end diastole and end systole to generate myocardial area per slice. The total myocardial volume (mm^3^) was calculated as the sum of myocardial area per slice (mm^2^) × number of slices × slice thickness (1 mm/slice). LV mass was calculated as myocardial volume times myocardial specific gravity (1.05 mg/mm^3^) and was presented as the average of mass independently calculated from end diastolic and end systolic measurements. Accurate measurement of MRI image analysis was confirmed in 3 ways: (i) no more than 5% difference in the myocardial mass calculated at end-diastole and end-systole; (ii) MR data was independently analysed by two investigators on 6 mice at both time points; and (iii) calculated mass showed good correlation with actual mass measured at autopsy (r = 0.91). To measure cardiac function, the LV chamber area outlined by the endocardial border for each slice at end diastole and end systole was measured and calculated for each slice at end diastole and end systole as LV volume per slice = LV area × slice thickness. The LV end-diastolic volume (LV-EDV) = ∑ LV volume per slice at end-diastole; and the LV end-systolic volume (LV-ESV) = ∑ LV volume per slice at end-systole. Functional parameters were calculated from these volumes as follows:

$$\text{Stroke volume}\left(\mathrm{SV}\right)=\mathrm{LV}‐\mathrm{EDV}-\mathrm{LV}‐\mathrm{ESV}\left(\mathrm{\mu l}\right).$$

$$\text{Ejection fraction}\left(\mathrm{EF}\right)\left(\%\right)=\left(\mathrm{SV}/\mathrm{LV}‐\mathrm{EDV}\right)\times 100.$$

Infarcted tissue is characterised by thinned, akinetic myocardium and infarct volume was calculated as a percentage of the left ventricular myocardium at end-diastole by measuring epicardial and endocardial infarct length (I_ep_ and I_en_) as a proportion of total epicardial and endocardial circumference (T_ep_ and T_en_) across all slices as previously described [8]. Infarct size (%) = 1/NSLICES Σ 1/2(I_ep_/T_ep_ + I_en_/T_en_) × 100.

### 2.4 Statistics

Multiple regression analyses were performed using STATA and R statistical software. Linear regression and correlation were calculated using Graphpad Prism 6. Statistical significance was assumed when p < 0.05.

## 3 Results

Cine MRI of infarcted heart shows the remodelled and enlarged LV chamber at 4 weeks post-MI, whilst the infarcted region can be recognised as a thinned area of akinetic myocardium (Fig. 2) [8]. Our aim was to determine which primary measure of cardiac function using MRI was best able to predict subsequent adverse outcomes and could be used as a predictive measure in future studies of therapeutic interventions. Week 1 was chosen for the baseline measurements for three reasons. First, some initial remodelling is needed to provide sufficiently early changes to permit analysis of therapies targeting further detrimental remodelling in future studies. Second, MR measurements at 1 week following acute MI have been shown to have greater predictive value for patient LV function than MR data at earlier time points [17]. Third, from the animal ethics (3Rs) perspective, one week allowed good recovery from the thoracotomy before mice were subject to transfer to the MR suite and further anaesthesia during the scans. The primary variables measured at week 1 were LV-EDV, LV-ESV, infarct size and LV-cardiac mass. Ejection fraction (EF) was also included as a key measure of cardiac functional, and represents a secondary variable calculated from LV-EDV and LV-ESV. The mice were then subjected to a second scan when the scar tissue had matured at 4 weeks post-MI. At this stage, increased LV-EDV and LV-ESV reflected adverse cardiac remodelling and chamber enlargement, whilst increased LV cardiac mass reflected cardiac hypertrophy and reduced ejection fraction corresponded to decreased cardiac function.

**Fig. 3:**
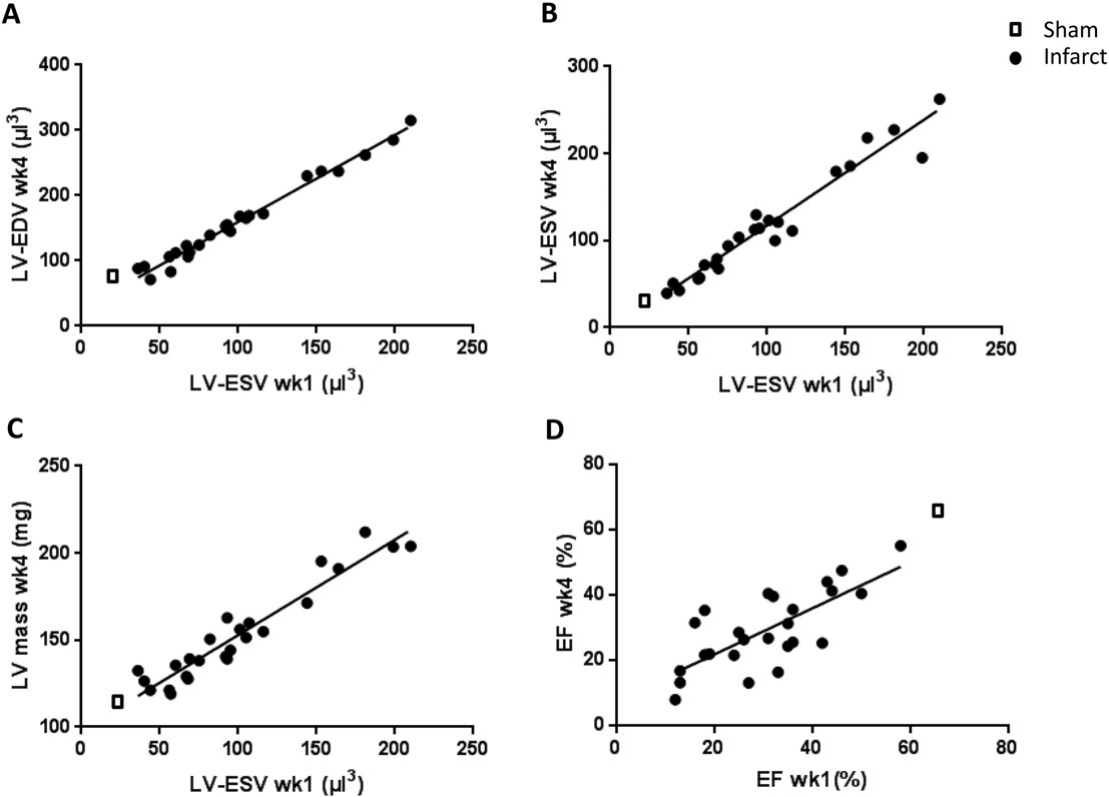
Linear regression analysis to evaluate LV-ESV at one week following MI as a predictor variable of adverse cardiac remodelling at four weeks post-MI. A: Linear regression analysis confirmed the strong linear relationship between LV-ESV_wk1_ and LV-EDV_wk4_ (r^2^ = 0.98; p < 0.0001) which is described by the equation: LV-EDV_wk4_ = 1.33(LV-ESV_wk1_) + 26 μl^3^. Each closed black circle represents one mouse following infarction and the open square represents the mean of 11 sham operated animals (standard error of the mean is small and fits within the size of the open square). B, C: LV-ESV_wk1_ showed a strong linear relationship with both LV-ESV_wk4_ (r^2^ = 0.94; p < 0.0001) and LV-mass_wk4_ (r2 = 0.92; p < 0.0001). D: LV-EFwk4 showed a reasonably good linear association with LV-EFwk1 (r^2^ = 0.74; p < 0.0001).

We first examined the relationship between each of five early variables at week 1 post-MI (LV-EDV_wk1_, LV-ESV_wk1_, LV-mass_wk1_, IS_wk1_ and LV-EF_wk1_) to each of the outcome measures at 4 weeks (LV-EDV_wk4_, LV-ESV_wk4_, LV-mass_wk4_, and LV-EF_wk4_) (Table 1). LV-ESV_wk1_ was highly correlated with LV-ESV_wk4_ (r = 0.97; p = 1.3 × 10^− 15^), and LV-ESV_wk1_ showed the highest correlation with LV-EDV_wk4_ (r = 0 99; p = 0.0000), a major readout of adverse left ventricular remodelling. In addition, LV-ESV_wk1_ was highly correlated with increased LV-mass_wk4_ (r = 0.96; p = 9.5 × 10^− 14^). However, LV-ESV_wk1_ did not show the highest correlation with LV-EF_wk4_ (r = − 0.64; p = 6.2 × 10^− 4^), and it was LV-EF_wk1_ that showed the highest correlation with LV-EF_wk4_ (r = 0.74; p = 2.5 × 10^− 5^) (Table 1). Linear regression analysis illustrates these key findings in Fig. 3.

**Table 1:** Correlation of cardiac measurements at one week following myocardial infarction with outcome measures at four weeks. Correlation coefficients (r) from univariate analysis (taking each week 1 variable against each week 4 outcome individually) show that left ventricular end systolic function at week one (LV-ESV_wk1_) is the best predictor variable for all outputs at week four except for ejection fraction (EF_wk4_), in which case EF_wk1_ showed the highest correlation. The highest r values for each outcome measure at week four are shown in bold, and all corresponding p values are in parentheses.

Variable	LV mass wk4	LV-ESV wk4	LV-EDV wk4	LV-EF wk4
LV mass wk1	0.87 (p = 1.8e − 8)	0.85 (p = 7.4e − 8)	0.89 (p = 2.7e − 9)	− 0.54 (p = 5.5e − 3)
LV-EDV wk1	0.94 (p = 4.7e − 12)	0.95 (p = 7.3e − 13)	0.98 (p = 0.0000)	− 0.59 (p = 1.7e − 3)
LV-ESV wk1	**0.96** (p = 9.5e − 14)	**0.97** (p = 1.3e − 15)	**0.99** (p = 0.0000)	− 0.64 (p = 6.2e − 4)
LV-EF wk1	− 0.83 (p = 2.5e − 7)	− 0.87 (p = 1.1e − 8)	− 0.86 (p = 4.6e − 8)	**0.74** (p = 2.5e − 5)
IS-wk1	0.85 (p = 6.6e − 8)	0.83 (p = 3.6e − 7)	0.87 (p = 1.9e − 8)	− 0.55 (p = 4.6e − 3)

Next, stepwise forward selection analysis was performed to determine which of the week 1 variables could best predict each of the different cardiac outcomes at week 4. Using this model, LV-ESV at week 1 was the strongest predictor variable for increased cardiac volume and mass measurements at week 4 (LV-EDV, LV-ESV and LV-cardiac mass) and no other variable made any significant contribution to the fit of the model. In contrast, LV-EF_wk1_ was the best predictor for LV-EF_wk4_ and inclusion of further week 1 variables (LV mass, LV-EDV, LV-EF or infarct size) did not significantly improve the fit to the model. These data confirm that LV-ESV_wk1_ is a very useful predictor variable for detrimental cardiac remodelling outcomes (increased LV-EDV, LV-ESV and LV-cardiac mass) at one month following infarction. Furthermore, LV-ESV_wk1_ is the most robust predictor of later adverse cardiac remodelling, accounting for 98% of the variability in LV-EDV_wk4_. Any future intervention able to reduce late adverse remodelling would be expected to significantly reduce the slope of the linear regression line described by the equation: LV-EDV_wk4_ = 1.33(LV-ESV_wk1_) + 26 μl^3^ (Fig. 3A).

## 4 Discussion

Reported benefits that attenuate LV dilatation and improve heart function in pre-clinical studies using mice are rarely translatable in clinical trials [6], an outcome that may partly reflect poor experimental design of the pre-clinical study. New consortia aim to address this problem by establishing shared methodologies to improve the translatability of preclinical studies using mice [6], [18]. However, in the vast majority of published studies investigating therapeutic interventions for MI in mouse models, the treated group is compared with the control group and mean outcomes of ejection fraction or scar size or left ventricle volumes are compared using Student's t-test. Frequently only one of these values is used (possibly selected as the only one showing statistical significance), and without the full dataset the conclusions may be misleading. Furthermore, the intrinsic variability in infarct size is likely to have a major impact on these analyses, and reduce the power of the study. Therefore in order to explore the predictive value of a mouse model of MI for pre-clinical studies in a way that accounts for variability in infarct size, we used cardiac MRI to evaluate heart remodelling responses in male mice following permanent ligation of the LAD coronary artery. We found that LV-ESV_wk1_ was a highly predictable measure of later adverse cardiac remodelling (LV-EDV_wk4_), with increased predictability over other MRI-derived measures of cardiac properties at week 1, including LV-EDV, heart mass and infarct size. The extent of adverse remodelling as judged by EDV_wk4_ was directly proportional to LV-ESV_wk1_ with small infarcts increasing by the same proportion as larger infarcts_._ This finding is in contrast to a previous study using female mice which observed a biphasic response, with smaller infarcts showing no progression [9]. This difference could be due to gender differences in the mice used for the studies. We used only male mice which show more inflammation and reduced reparative fibrosis during infarct healing than females [10], [11], [12], [13], and this may explain why even small infarcts still showed some adverse remodelling in our study. In contrast, the reduced inflammation in females and increased reparative fibrosis may explain the lack of progression of small infarcts previously reported [9]. Alternatively, the direct linear regression relationship between early and later points in our study may be because the initial adverse remodelling events are better established at the 1 week post-MI time point, compared with the 2 day time point used by Protti et al. [9]. Although stepwise forward analysis did not reveal any additional contribution of infarct size at week 1 over LV-ESV_wk1_ to predict adverse remodelling, univariate correlation analysis showed that infarct size measured by simple manual tracing of thinned akinetic myocardium [8] significantly correlated with LV-EDV_wk__4_ outcomes (r = 0.87; p < 0.0001). Furthermore, laboratories without access to a pre-clinical MRI facility could potentially use evaluation of end diastolic diameter (EDD) by echocardiography as an alternative predictive measure at early time points post-MI, although caution is needed because this technique is open to more subjective interpretation than MRI.

Progressive ventricular dilatation after myocardial infarction is part of a complex cardiac remodelling process involving changes in mass, shape and volume [3]. Limited left ventricular dilatation can help to retain cardiac function and is therefore compensatory, but further dilatation can result in severe LV dysfunction and heart failure. Patients with major LV remodelling have worse prognosis than patients with smaller changes [2], [3] and even small reductions in adverse LV remodelling can reduce the risk of progression to heart failure [19], [20]. Although EF is the most frequently used measure of heart function based on its clinical importance, it reflects a combination of heart dilatation and myocardial contractile function, making it more challenging to interpret than the simpler mathematical measurement of left ventricle enlargement over time to quantify adverse cardiac remodelling.

### 4.1 Study limitations

Limitations of using mouse models of clinical MI include the different coronary anatomy of mice and humans [21]. LAD ligation in mouse occludes the vascular supply to much of the free wall of the left ventricle, sparing the septum and right ventricle. Mice have a distinct septal coronary artery from the ostium above the right sinus or as a branch of the right coronary artery, resulting in sparing of the interventricular septum after occlusion of the LAD [21]. In contrast, the vascular network of the interventricular septum in human hearts is frequently supplied by branches from the LAD which means that myocardial infarction due to occlusion of the LAD frequently affects the septal myocardium. A further limitation is the use of healthy young adult mice in these types of study, whereas older mice with atherosclerosis (e.g. *ApoE* mutant mice) would provide a more clinically relevant study population. We also acknowledge that measurement of cardiac function at 1 week post-MI would not provide a useful baseline for measuring early cardioprotective treatments, e.g. ischaemic pre-conditioning where reproducible benefit can be seen in pre-clinical models at 48 h post-MI [18]. However, it is an ideal time point for testing therapies designed to reduce progressive adverse remodelling where patient risk is ascertained by MR following MI. In the clinical setting, MR measurements at 1 week following acute MI have greater predictive value for patient LV function than MR data acquired at day 2 [17].

## 5 Conclusion

Our findings suggest that MRI evaluation of LV-ESV at one week following MI in mouse models can be used to accurately predict subsequent adverse remodelling and therefore represents a reliable baseline measure to facilitate a more robust analysis of interventions to ameliorate adverse progression of cardiac remodelling following MI.

## Conflicts of interest

The authors report no relationships that could be construed as a conflict of interest.

## Grant funding support

This work was funded by a programme grant from the British Heart Foundation (RG/12/2/29416). STC was funded by an Intra-European Marie Curie Fellowship (329813 VASC-GEN) and JES is a British Heart Foundation Senior Basic Science Research Fellow (FS/11/50/29038).
